# The effect of human-specific genetic variants on neuronal spinogenesis

**DOI:** 10.3389/fgene.2026.1786287

**Published:** 2026-06-03

**Authors:** Nicolás Matías Rosas, Anna Szombathy, Kinga Szigeti

**Affiliations:** Department of Neurology, State University of New York at Buffalo, Buffalo, NY, United States

**Keywords:** actin cytoskeleton, dendritic spine, human brain, human-specific genes, neuropsychiatric disease

## Abstract

Fundamental morphological and functional differences between the brains of animal models and humans are at least partially related to human-specific genes and genetic variants. As one of the structural underpinnings of brain function is the dendritic spine, we systematically queried a curated list of human-specific genes and genetic variants. We found that with the current knowledge base, 4.3% are linked to the dendritic spine. Functionally these genes converge on the cytoskeleton, Ca^2+^ signaling, small GTPases, NMDAR, and WNT signaling and trafficking suggesting human specific modification of canonical pathways. Significant gaps in knowledge are identified and concerted efforts are needed. Understanding human-specific genetic contributions to the unique features of the human brain will address existing translational gaps and facilitate the development of successful treatments for neuropsychiatric disorders, advance environmental neuroscience for early childhood intervention and environmental risk reduction in aging and dementia.

## Human-specific genetic variants

**FIGURE 1:**
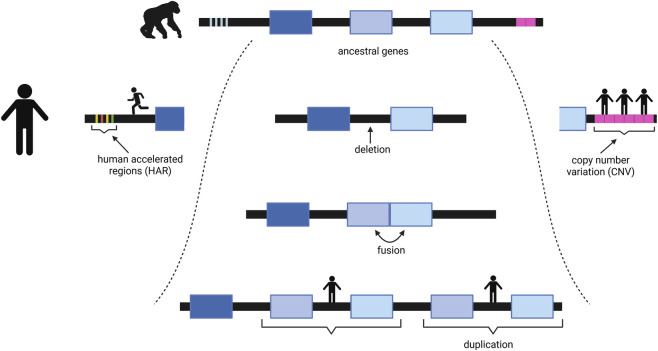
Genomic variations contributing to human-specific dendritic spine development. This diagram illustrates evolutionary genetic modifications including deletion, fusion and duplication that influence dendritic spine morphology and synaptic plasticity in the human brain. Blue pattern blocks represent ancestral gene segments. Human Accelerated Regions (HARs) denote genomic segments that have evolved rapidly and are related to neurodevelopmental regulation. Copy Number Variation (CNV) can contribute to gene dosage, thereby modulating the human brain (Created in BioRender.com).

Human-specific genetic variants are defined as genes and genetic polymorphisms that have evolved uniquely in *Homo sapiens* and are absent in other species. These have emerged through multiple genetic mechanisms including gene duplications, novel gene regions, retrotransposon-mediated events, fixed single nucleotide variants (SNVs), and sequence and structural modifications (Bitar et al., 2019; Soto et al., 2025; Charrier et al., 2012; Ihnatovych et al., 2024; Lu et al., 2019) (Figure 1). Duplications of existing genes lead to dosage effect (Bitar et al., 2019; Soto et al., 2025), and often one of the copies further evolves and harbors fixed genetic variations that acquire new functions leading to complex genetic mechanisms (Charrier et al., 2012). Partial duplications and complex genetic rearrangements result in novel sequences and fusion genes (Ihnatovych et al., 2024). Fixed sequence modifications in the human lineage can lead to gain of function or evolutionarily beneficial hypomorphism or loss of function (Krause et al., 2007; Szigeti et al., 2020; Szigeti et al., 2024; Szigeti et al., 2023).

In addition, Human Accelerated Regions (HARs) play a significant role in brain evolution, fine-tuning gene expression levels and influencing neuronal development and communication (Pal et al., 2025). HARs are highly conserved across vertebrates but show significant differences in humans, suggesting they play a crucial role in shaping human brain complexity and cognitive abilities (Pal et al., 2025; Ruiz-Jimenez and Santpere, 2025) (Figure 1). Recently, human-specific expanded short tandem repeats (heSTRs) were found to work as neuron enhancers, facilitating chromatin accessibility and increased transcription factor binding in human neurons (Liu and Tian, 2025). These findings underscore the importance of regulatory sequence evolution in the emergence of human brain complexity.

Human-specific genetic variants have been pivotal in our adaptation to environmental changes such as immune challenges and dietary shifts, which have been crucial for human survival and thus were subject to selective pressure that increased allele frequency (Bitar et al., 2019; Patin and Quintana-Murci, 2025; Bragazzi et al., 2024). Ongoing exposure to agro-environmental effects may also drive selective pressure (Vatsiou et al., 2016). Evidence of genetic mixing between ancient populations suggests that modern humans are the result of complex genetic evolution involving multiple ancestral lineages (Cousins et al., 2025). Full genome sequences have revealed that two ancestral populations diverged around 1.5 million years ago and later reconnected about 300,000 years ago, contributing significantly to the genetic makeup of modern humans (Cousins et al., 2025). These findings further illustrate the dynamic nature of human genetic evolution.

Recent studies have identified a substantial number of high-confidence human-specific genomic variants, primarily associated with protein-coding regions (Bitar et al., 2019; Soto et al., 2025). Annotation reveals that these genes play significant roles in brain, immune, and metabolic adaptations (Bitar et al., 2019; Soto et al., 2025). A subset of these genes is specifically associated with neural activity and the expansion of the human cortex in dynamic spatial and temporal contexts (Bitar et al., 2019; Soto et al., 2025; Tynianskaia and Heide, 2024).

Human-specific variants can be categorized as obligatory or facultative (Bitar et al., 2019; Soto et al., 2025). Obligatory genetic variants are those essential for the development and maintenance of the human phenotype; they are typically conserved across populations and underpin fundamental biological processes such as cellular function, neurodevelopment, and survival (Bitar et al., 2019; Soto et al., 2025). These variants are crucial for the structural and functional integrity of the brain and other organ systems. In contrast, facultative genetic variants contribute to the diversity of brain function and modulate susceptibility to neurological and psychiatric disorders. These variants are not required for basic phenotypic expression but influence the genetic background that interacts with environmental factors to determine disease risk and cognitive traits.

## Technologies for detecting human-specific genetic changes

Advancements in genomic technologies and sequencing of genomes from modern and archaic hominins, great apes, and other primates have greatly enhanced our awareness of human-specific genetic changes (Soto et al., 2025; Pollen et al., 2023). Concerted efforts such as the T2T sequencing project constantly improve the reference genome to facilitate mapping (Soto et al., 2025). Long-read sequencing technologies, such as Pacific Biosciences’ Single Molecule Real-Time (SMRT) sequencing and Oxford Nanopore Technologies’ nanopore sequencing, have significantly enhanced our ability to detect and characterize genomic and transcriptomic variants with high resolution. Unlike short-read sequencing, which often fails to resolve repetitive regions, structural variants, and complex genomic rearrangements, long-read platforms generate reads spanning tens to hundreds of kilobases, enabling the accurate identification of large insertions, deletions, inversions, and translocations (Soto et al., 2025; Romagnoli et al., 2023; Zamora-Canovas et al., 2024). These technologies also facilitate haplotype phasing, allowing for the reconstruction of allele-specific expression and inheritance patterns (Zhao et al., 2025; Diaz-Riano and Duitama, 2025). In transcriptomics, long-read sequencing captures full-length RNA molecules, thereby revealing alternative splicing events, fusion transcripts, and isoform diversity that are often missed by short-read methods (Monzo et al., 2025a; Oikonomopoulos et al., 2020). Long-read sequencing also supports direct detection of epigenetic modifications, contributing to a more comprehensive understanding of gene regulation and expression (Bein et al., 2025). Overall, these advances are transforming genomics and transcriptomics by offering a more nuanced and complete view of genetic variation and transcriptome complexity.

Despite their transformative impact on genomics and transcriptomics, long-read sequencing technologies face notable limitations in resolving complex human-specific variants. These include elevated raw error rates, particularly in single nucleotide variant detection, and reduced throughput and cost-efficiency compared to short-read platforms, which can hinder large-scale applications (Amarasinghe et al., 2020). Coverage biases persist in challenging genomic regions such as centromeres, telomeres, and segmental duplications—areas often rich in human-specific structural and regulatory variation (Soto et al., 2025; Mantere et al., 2019). In transcriptomics, long-read methods may struggle with low-abundance isoforms and complex splicing patterns, limiting full transcriptome resolution (Monzo et al., 2025a; Monzo et al., 2025b). Additionally, phasing across large genomic distances remains difficult, and reliance on incomplete reference genomes can obscure population-specific variants (Zhao et al., 2025). Finally, bioinformatics tools for long-read data are still evolving, which can affect the accuracy and consistency of variant detection and interpretation (Amarasinghe et al., 2020).

New molecular and cellular approaches, including single-cell sequencing, genetic manipulation, and human-specific stem cell and organoid culture have provided the technical tools to fully characterize molecular mechanisms (Pollen et al., 2023; Menche and Farin, 2021). Eventually human clinical studies will be necessary to validate the findings from these new approaches and to fully understand their implications in brain, immune and metabolic function in a complex host.

## Human-specific differences in dendritic structure and function

Human-specific structural and functional differences highlight the evolutionary adaptations of dendritic architecture to meet the demands of our species’ nervous system. Quantitative genetic gains alone cannot fully explain the divergence between primate and human brain function; instead, qualitative genetic evolution, leading to human-specific genes and variants, likely provides a more in-depth understanding. To address this, we reviewed current knowledge on human-specific genetic variants in the context of brain structure and function, focusing on the dendritic spine, a key structure for neural development.

The human brain exhibits a uniquely refined structural organization characterized by complex neuronal morphology and cortical architecture. Human pyramidal neurons display extensive dendritic arborization, with increased perisomatic branching and thicker dendrites that enhance long-range connectivity across cortical networks (Kanari et al., 2025). These dendrites harbor thicker and longer spines, promoting synaptic strength and plasticity. Unlike mice, the human brain has achieved higher-order integration by enhancing the structural complexity of individual neurons rather than increasing neuron number (Kanari et al., 2025; Masoli et al., 2024). The topological complexity of dendritic trees correlates with memory capacity, expanding the combinatorial space for synaptic input processing (Masoli et al., 2024). Together, these features illustrate an evolutionary strategy in which single-neuron complexity underpins human cognitive abilities.

Dendritogenesis arises from the interplay of extrinsic and intrinsic mechanisms (Lin et al., 2020; Hamad et al., 2023). Extrinsic cues such as adhesion molecules and secreted factors shape dendritic patterning, while intrinsic programs regulate transcriptional control and lipid metabolism for membrane expansion (Lin et al., 2020). Growth factors including BDNF and NGF activate MAPK/ERK, PI3K/Akt, and mTOR pathways essential for dendritic growth (Moya-Alvarado et al., 2023). Additional regulators, such as Rho GTPases, calcium signaling, and cytoskeletal proteins, fine-tune dendritic dynamics and synaptic connectivity (Lavanderos et al., 2020; Li et al., 2021). These processes collectively contribute to the increased arborization and node density observed in human cortical neurons, supporting advanced cognitive functions like learning and memory consolidation (Kanari et al., 2025).

Dendritic spines are actin-rich protrusions that compartmentalize biochemical signaling and serve as principal postsynaptic sites for excitatory neurotransmission. Their formation is initiated by glutamatergic activity, leading to calcium influx through NMDA receptors (Runge et al., 2020) and activation of CaMKII, Ras–MAPK, and mTOR pathways that regulate actin remodeling and local protein synthesis (Jedrzejewska-Szmek and Blackwell, 2019). Local translation of synaptic proteins such as PSD-95, ARC, and HOMER1 ensures spine stabilization and efficacy (Swanger and Bassell, 2013). The postsynaptic density (PSD) anchors AMPA and NMDA receptors along with scaffolding proteins, while metabotropic glutamate receptors (e.g., mGluR5) mediate slower G-protein–coupled signaling essential for plasticity mechanisms such as LTP and LTD (Bosch et al., 2014).

Although spinogenesis is evolutionarily conserved across species, involving actin regulators like Cofilin and Arp2/3 and translational control factors (Ben et al., 2020; Ono et al., 1999; Norris et al., 2009; Rust, 2015; Spence et al., 2016), the timing and extent of synaptogenesis and pruning differ markedly between humans and other primates. Human pyramidal neurons generally possess a higher total number of spines per cell, enabling greater input integration, although regional variation exists—for example, there is lower spine density in the human visual cortex compared to the same location in mice (Benavides-Piccione et al., 2025). Additionally, in humans, synapse proliferation and pruning occur heterogeneously across cortical regions, with prefrontal synaptogenesis peaking in mid-childhood and pruning extending into adulthood, unlike the more synchronized maturation seen in rhesus and macaque monkeys (Huttenlocher and Dabholkar, 1997; Bianchi et al., 2013).

Structurally, human dendritic spines are larger, with elongated necks and broader heads than those of rodents (Ofer et al., 2022). Quantitative comparisons show that human spine head volume is roughly twice that of mouse (0.32 vs. 0.146 μm^3^), with proportionally longer necks and wider diameters. Human synapses also feature more complex PSDs and higher densities of synaptic vesicles and active zones, enhancing synaptic efficacy (Zhang et al., 2021; Heckmann and Pauli, 2022; Italia et al., 2022; Purkey and Dell'Acqua, 2020; Chowdhury and Hell, 2019). Despite general conservation of synaptic proteins, key PSD components related to structural plasticity differ between species (Bayes et al., 2012; Koopmans et al., 2018), and active zones and vesicles are larger in humans, reflecting enhanced signal modulation (Palma-Barqueros et al., 2021; Molnar et al., 2016).

While the molecular machinery governing dendritic spine formation is largely conserved, the structure, timing, and complexity of spine maturation displays remarkable species-specific patterns. We therefore hypothesize that genes harboring human-specific genetic variants converge into major conserved molecular pathways that regulate dendritic spine formation and remodeling. These pathways are critical in shaping the unique architecture and plasticity of human neural circuits and may ultimately contribute to advanced cognitive and behavioral capacities. To evaluate this, we conducted a review that synthesizes curated evidence of genetics, molecular neuroscience, and comparative biology to classify genes with human-specific variants and asses their relevance to dendritic spine biology.

## Methods

The curated list of 856 high-confidence human-specific genomic variants published by Bitar et al. was evaluated for relevance to the human dendritic spine (Bitar et al., 2019). Each gene was individually queried using Microsoft Copilot to asses potential involvement in the following: cytoskeleton function, neuron function, cell structure (especially cytoskeleton of neurons or dendritic spine), and neuronal pathology or disease. To avoid possible variability and differences in the output when using a large input prompt, queries were carried for each gene individually. The AI prompt used can be found in Supplementary Material.

We performed data collection in 3 phases:

1. AI-assisted screening phase: Microsoft Copilot was used to identify genes with potential relevance to dendritic spine function.
2. Manual validation phase: Genes identified as relevant or inconclusive were manually reviewed, validated, and searched in both OpenEvidence and PubMed to identify peer-reviewed cited references.
3. Manual reference curation phase: All identified peer-reviewed references were reviewed for relevance and manually curated into tables. The reference lists of each citation were also reviewed to expand the citation base.

We used the following inclusion criteria for the tables: (i) original research article; (ii) human specific variants or ancestral gene incorporated into the experimental design; and (iii) dendritic spine specific experiments or reported human phenotype (e.g., cognitive, MRI).

The final dataset was independently reviewed by at least two authors, and all entries were cross-validated. Data reflects current knowledge as of 21 October 2025. Inclusion required direct verification in primary peer-reviewed literature; therefore, AI-generated false positives were not incorporated into the final dataset. However, as the initial step relied on AI-assisted screening, relevant genes not captured during the first phase may not have progressed to the manual review, therefore some false negatives cannot be excluded.

## Human-specific differences in dendritic structure and function

Most of the genes listed are linked to neuropathological conditions although the association is not uniform. Some genes are broadly implicated in neurodevelopmental or neurological disease vulnerability, whereas others have particular human-specific substitutions, duplication, or regulatory changes with ties to specific diseases (Supplementary Table S1).

Genetic variations in several genes in the dataset are associated with major neurodevelopmental disorders, including autism spectrum disorder, intellectual disability, epilepsy, and language impairments. For example, three individuals with microdeletions variants in *AGAP1* had intellectual disability (3/3), autism (3/3), dystonia (1/3), abnormalities of brain maturation (1/3), growth impairment (2/3) and facial dysmorphism (2/3) (Lewis et al., 2023). Similarly, *CNTNAP2* variants are associated with autism spectrum disorder, intellectual disability and language disorders, and a single nucleotide polymorphism in gene sequence was associated with autism (Jang et al., 2023; D'Onofrio et al., 2023; Smogavec et al., 2016; Rodenas-Cuadrado et al., 2014). Other genes, such as *NLGN4X* and *NBEA*, show similar associations with autism, seizures and broader neurodevelopmental syndromes such as intellectual disability or learning disorders (Nguyen et al., 2020; Lawson-Yuen et al., 2008; Mulhern et al., 2018; Volders et al., 2011; Miura et al., 2021; Jacobsen et al., 2015).

Clinical neuropsychiatric phenotypes, including schizophrenia, bipolar disorder, anxiety and mood disorders, are also linked to the human variants. For instance, *CDH12* is associated with bipolar disorder and schizophrenia (Redies et al., 2012), while *CRHR1* is linked to major depressive disorder, panic disorder and anxiety (da Silva et al., 2016; Weber et al., 2016; Binder and Nemeroff, 2010). Several genes with human-specific substitutions–such as *DXDC1, CHRFAM7A* and *GRIN3A*–also show associations with schizophrenia and psychiatric phenotypes (Ihnatovych et al., 2024; Martin et al., 2018; Takata et al., 2013).

*APOE*, *SEPTIN7* and *MAPT* have been linked to neurodegenerative diseases. *APOE* is the most prominent example, where its alleles are strongly associated with Alzheimer’s disease (Fortea et al., 2024; Heise et al., 2024). Changes in *SEPTIN7* expression are also linked to Alzheimer’s disease and schizophrenia (Wang X. et al., 2018), while *MAPT* is associated with dementia, corticobasal degeneration and increased Alzheimer’s disease risk (Greaves and Rohrer, 2019; Forrest et al., 2018; Zhang et al., 2017; Tahir et al., 2000; Buchholz and Zempel, 2024).

Finally, several genes are associated with rare neurological syndromes or brain structural abnormalities. *CDK5RAP2* pathogenic variants cause microcephaly and developmental defects the hypothalamus, retina and cochlea (Nasser et al., 2020; Rimol et al., 2010; Erdogan et al., 2025; Kraeme et al., 2011). *OPHN1* variants lead to X-linked intellectual disability and cerebellar hypoplasia (Bergmann et al., 2003; Chabrol et al., 2005; Santos-Reboucas et al., 2014).

These human central nervous system clinical phenotypes associated with a pathogenic variation of the ancestral gene or the human specific variant provide experimental proof that these genes are relevant for human brain function and disease susceptibility.

## Human-specific genetic variants annotated to the dendritic spine

**FIGURE 2:**
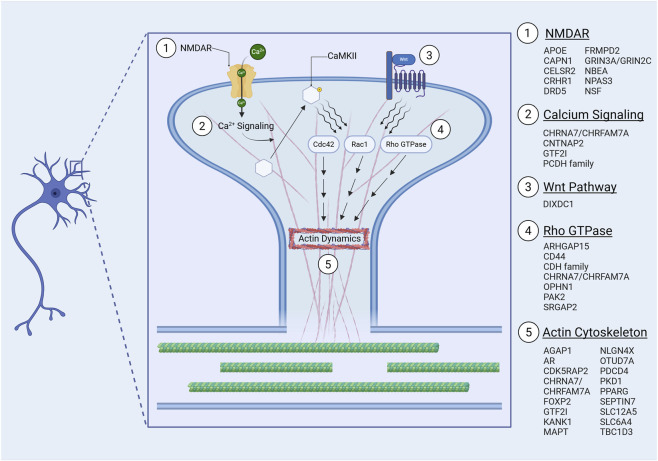
Dendritic spine pathways influenced by human-specific genes. Human-specific genes impacting spinogenesis fall into five major mechanistic categories, which are (1) NMDA receptor (NMDAR) modulation, (2) calcium signaling, (3) the Wnt pathway, (4) Rho GTPases, and (5) the actin cytoskeleton. This schematic highlights where each of those mechanisms takes place along the dendritic spine and denotes the human-specific genes relevant to each mechanism. Some genes are only known to exert effects through a single mechanism, while others have roles at multiple levels of regulation and thus appear more than once. The figure also demonstrates the interconnectedness of these pathways in governing dendritic spine structure. The NMDAR is heavily involved in calcium signaling, which then exerts effects on downstream GTPases through CaMKII to influence actin dynamics. Meanwhile, the Wnt pathway also has interplay with GTPase activity to influence the actin cytoskeleton. The effect of human-specific genes at each of these levels of regulation culminates to form a uniquely human dendritic spine (Created in BioRender.com).

We found that while the ancestral gene is often well characterized, the implications of human-specific sequence alterations have only been investigated in a limited number of genes, suggesting that our current knowledge is just the tip of the iceberg. Thus, we extended the curated annotation to genes where the ancestral gene has been implicated in dendritic spine biology albeit the human variant’s specific effect is unknown. 153 (18%) of the human-specific genes and variants are related to the cytoskeleton and 36 (4%) have established connections to the dendritic spine (Figure 2). The marked overlap between the cytoskeleton and dendritic spine (78% of dendritic spine annotated genes are also annotated to the cytoskeleton) suggests that a large proportion of the cytoskeletal genes that are not yet annotated to the dendritic spine will play a role if experimentally studied.

Mapping human-specific variants linked to dendritic spines onto conserved pathways revealed that these variants are not randomly distributed across cellular function. Instead, they revealed a pronounce clustering in molecular modules that regulate cytoskeleton remodeling, Ca^2+^-dependent signaling, RhoGTPase activity and endosomal trafficking. This pattern proves that these pathways represents recurrent evolutionary targets and may have contributed to enhanced structural plasticity and spine complexity characteristic of the human brain.

### Genes impacting the cytoskeleton

Since the cytoskeleton is the structural underpinning of the dendritic spine, it is not surprising that among the identified pathways, cytoskeleton regulation was the most represented category for human-specific genes and variants annotated to the dendritic spine. Several genes converge on actin dynamics (*CDK5RAP2*, *KANK1*, *PPARG, OTUD7A, PKD1*), whereas others influence microtubule changes (*MAPT*, *SLC12A5*). Together, all influence the dendritic spine and synaptic structure (Figure 2). CDK5RAP2 is a regulator of cyclin-dependent kinase 5 (Cdk5), which is involved in actin dynamics and is necessary for remodeling and maintenance of dendritic spines (Zaqout et al., 2019; Jin et al., 2016). Rosiglitazone (best known for its application in diabetes) is an agonist of PPARG that increases dendritic spine density by regulating the actin cytoskeleton although its mechanism has not been elucidated (Brodbeck et al., 2008). Interaction of KANK1 with Talin-1, a component of focal adhesions that links integrins with the actin cytoskeleton, was related to the density and formation of mushroom-type spines in rat hippocampal neurons (Sun et al., 2025). The microtubule associated protein Tau (MAPT) is a substrate for a large number of kinases that regulate its binding to microtubules and actin; this binding is key for the localization of tau in post synaptic spines. Phosphorylation of MAPT regulates the spine density, length and area by modifications of Tau binding to microtubules (Ittner and Ittner, 2018; Xu et al., 2014). *SLC12A5* is a gene that encodes the neuronal potassium-chloride co-transporter 2 (KCC2) with different expression and functions during development. During hippocampal development, KCC2 decreases spine density with a mechanism dependent on BDNF expression and correct potassium-chloride transport activity. Oppositely, expression of KCC2 in cortical neurons increases spine formation independently of its transporter activity and functioning as a scaffold protein to interact with F-actin (Puskarjov et al., 2014; Awad et al., 2018). OTUD7A regulates spine density likely through indirect interaction with ankyrins that connect the membrane with the cytoskeleton (Yin et al., 2018). PKD1 directly binds N-cadherin increasing surface localization triggering its phosphorylation to promote linking to actin cytoskeleton and formation of spines and functional synapses (Li et al., 2019).

Taken together, the genes in this category suggest that cytoskeleton-mediated structural plasticity constitutes a major evolutionary target of human-specific variants.

### Genes modulating Ca^2+^ signaling

Calcium-dependent pathways that regulate actin-binding proteins affect the humanized actin cytoskeleton. *CNTNAP2* and *PCDH* gene family are linked to Calcium-dependent pathways. In addition to its function as a transcription factor, CNTNAP2 is a scaffold protein that interacts with Calcium/calmodulin-dependent Serine protein Kinase (CASK) to regulate spine density through the stabilization of newly formed dendritic spines not affecting the ratio of formation of new spines (Gdalyahu et al., 2015). PCDH15, PCDHA2, PCDHA5, PCDHB10, PCDHB11-15 and PCDHB6 are part of the protocadherins (*PCDH*) family that binds calcium and are thought to interact with scaffold proteins that link them to actin-regulatory pathways to regulate dendritic spines (Pancho et al., 2020). Within this family, PCDHγ cluster and PCDH8 were related to the density of dendritic spines and PCDH7 was related to maturation (Molumby et al., 2017). This patters proves that human evolution may have modified not only the structural features of dendritic spines but also the Ca^2+^-dependent pathways related to maturation and stabilization. This calcium-regulated mechanisms form the second major gene cluster of human-specific variants.

### Genes influencing Rho GTPases

Human-specific genes linked to Rho and Rab GTPases converge on the common function of controlling the timing and structural remodeling of dendritic spines. Because Rho GTPases are upstream regulators of the actin cytoskeleton, these variants may provide the evolutionary route to modify spine maturation without altering initial spine formation machinery. *CHRFAM7A* provides a striking example of a human specific fusion gene that has been characterized for its role in dendritic spine morphology. *CHRFAM7A* is a fusion gene between *CHRNA7*, the subunit of the α7 nicotinic acetylcholine receptor (α7 nAChR) and *FAM7A/ULK4. CHRFAM7A* has two alleles, direct and inverted, thus it is not just human-specific, but divides the human population into 3 genotype groups with 25%–50%–25% split (Szigeti et al., 2020). Both alleles underwent strong selective pressure and are present in 99.7% of the human population; of note, null humans carrying none of the alleles are phenotypically normal suggesting that the alleles are not developmentally necessary (Szigeti et al., 2020). Translated CHRFAM7A from the direct allele humanizes the α7 nicotinic acetylcholine receptor (α7 nAChR), leading to a human-specific shift of the calcium (Ca^2+^) reservoir from the extracellular space to the endoplasmic reticulum (Szigeti et al., 2024; Szigeti et al., 2023). This shift in Ca^2+^ dynamics influences the activation of the small GTPase RAC1 (Szigeti et al., 2024; Szigeti et al., 2023). RAC1 activation by CHRFAM7A affects the organization and dynamics of the actin cytoskeleton within dendritic spines (Szigeti et al., 2023). As RAC1 activation leads to the reorganization of the actin cytoskeleton, it results in a phenotypic transition from filopodia to lamellipodia (Szigeti et al., 2024; Szigeti et al., 2023). This transition corresponds to the shift from dendritic filopodia to mature dendritic spines with distinct head and neck structures (Szigeti et al., 2023).

Clinical research involving 46 subjects who underwent neuropsychological tests and structural and functional MRI scans showed that carriers of the direct allele had smaller brain volumes and higher visual network connectivity (Jakimovski et al., 2024). *CHRFAM7A* direct allele carriers exhibited better cognitive performance, particularly in processing speed, visual learning, and memory. These findings suggests a link between the *CHRFAM7A* direct allele and more efficient brain function (Jakimovski et al., 2024). A human isogenic iPSC model confirmed that the inverted allele is not translated and the function of the inverted allele thus remains elusive (Ihnatovych et al., 2024).

A second convergent mechanism involves the modulation of synaptic maturation timing. *TBC1D3* is located on chromosome 17q12 and has roles in endosomal fusion, neuronal maturation and morphology, and tumorigenesis. TBC1D3 modulates the timing of dendritic spine development. At early stages of differentiation TBC1D3/MICAL1 promotes dendritic formation by increasing actin dynamics and at later stages, the protein complex regulates gene expression for synaptic maturation through chromatin remodeling factor ATRX (Dong et al., 2024). In humans, there are multiple paralogous copies of the gene, and there is significant copy number variation between humans. Expression patterns also show significant variation between individuals and tissue types (with highest expression in the testis and brain) (Hodzic et al., 2006; Guitart et al., 2024).

TBC1D3 functions as a GTPase activating protein for RAB5A, which is involved in early endosome fusion. It is also thought to function as an oncogene, as transformed cells were tumorigenic in mice, and the gene has previously been associated with prostate cancer (Hodzic et al., 2006). *TBC1D3* has been implicated in the evolution of cortical folding in humans and primates, and it also has notable effects on the neuronal synapse and dendrites. Knockdown of *TBC1D3* in human PSC-derived cortical neurons revealed precocious spine maturation and reduction of dendritic growth. *TBC1D3*-KO neurons exhibited marked reduction in total dendritic length and branching. Spine density in *TBC1D3*-KO neurons reached a peak 2–3 weeks earlier and was significantly lower than that of control neurons. Moreover, *TBC1D3*-KO neurons show a reduction in amplitude and frequency of spontaneous excitatory postsynaptic currents. In contrast, when human TBC1D3 was expressed in the mouse cortex, synaptic maturation was prolonged and dendritic growth was enhanced. MICAL1 was identified as a binding protein to TBC1D3 and dendritic arborization and spine maturation is dependent on this interaction (Dong et al., 2024). TBC1D3 seems to mediate dendritic growth via an interaction with MICAL1 to enhance actin dynamics. TBC1D3 and MICAL1 also interact with the chromatin remodeling factor ATRX to prolong spine and synapse maturation (Frasa et al., 2012). These results suggest that hominoid-specific *TBC1D3* and the human-specific expansion both play a decisive role in neuronal morphology which may have facilitated the evolution of more complex brain architecture in hominoids and humans.

The *SRGAP2* gene family has undergone a series of human-specific duplication events that are hypothesized to have contributed to the evolution of the human neocortex and associated cognitive functions (Martins et al., 2018). The ancestral gene, *SRGAP2A*, is conserved across mammals and plays a critical role in neuronal migration and dendritic spine maturation by promoting the formation of fewer, more stable synaptic connections.

In the human lineage, *SRGAP2A* underwent a segmental duplication approximately 3.4 million years ago, giving rise to *SRGAP2B*. A subsequent duplication of *SRGAP2B* produced *SRGAP2C* around 2.4 million years ago, followed by a third duplication event that generated *SRGAP2D*. Among these, *SRGAP2C* is the most functionally significant and is fixed in modern humans, while *SRGAP2B* and *SRGAP2D* exhibit copy number variation (Dennis et al., 2012).

*SRGAP2C* encodes a truncated protein that acts as a dominant-negative inhibitor of SRGAP2A, delaying dendritic spine maturation and increasing spine density (Charrier et al., 2012). This neotenous effect on synaptic development is thought to enhance synaptic plasticity and may have facilitated the emergence of higher cognitive functions in early Homo species. The timing of these duplications coincides with key milestones in hominin evolution, including increased brain size and the appearance of tool use.

Although the ancestral gene is well characterized, the functional relevance of the human-specific variants remain unknown for variants *ARHGAP15*, *CDH12*, *CD44, OPHN1, PAK2* and *AGAP1,* representing a gap in knowledge of how human evolution reshaped control of spine structure. ARHGAP15 is a RAC1-specific GTPase-activating protein (GAP) that is directly involved in spine density without an effect on maturation (Zamboni et al., 2016). CDH12 is a cadherin that activates the GTPases RAC1 and CDC42 to regulate cytoskeleton organization during development. CDH12 belongs to the type II cadherin family which has been related to formation of mature spines in mice (Bian et al., 2015; Basu et al., 2017). Cleavage of hyaluronan receptor Cd44 activates Cdc42 and Rac1 GTPases to increase dendritic spine head and length (Ro et al., 2016). OPHN1 regulates Rho GTPase activity, controlling the maturation of spines and is necessary for stabilizing/maintaining spine structure (Khelfaoui et al., 2007; Nadif et al., 2009). Pak2, a protein kinase that is a downstream target of Cdc42 and Rac1, is critical for spine density through the LIMK1-cofilin pathway (Wang Y. et al., 2018; Zhang et al., 2022). AGAP1 regulates small GTPases involved in endosomal trafficking and fine-tuned expression of AGAP1 is necessary to maintain spine density and morphology (Arnold et al., 2016).

Collectively, this category indicate that human-specific modulation of GTPases converges in extending or reshaping the maturation of dendritic spines to enhance adaptative synaptic remodeling.

### Human-specific genetic variants modulating the post synapse

Human-specific variants that are subunits or regulate NMDAR and AMPAR define the dendritic spine on the postsynaptic side. GRIN3A and GRIN2C are both subunits of NMDA receptors. GRIN3A was reported to be involved in the rate of mushroom spine elimination without affecting the elimination of filopodia and stubby (Kehoe et al., 2014). CELSR2 is a cadherin receptor that modulates the activity of NMDAR and has a role in establishing mature spines (Chen et al., 2024). FRMPD2 is a scaffold protein that binds directly to the GluN2A subunit of NMDA receptors and modulates the proportion of mature and immature spines (Lu et al., 2019). Corticotropin-releasing hormone receptor 1 (CRHR1) regulates CRH-mediated dendritic spine loss through NMDA activity which was proposed as a stress adaptation mechanism (Andres et al., 2013). Dopamine receptors, including the human-specific DRD5, are localized to dendritic spines and have a role in the maintenance of spines through NMDA and AMPA mediated PKA phosphorylation (Fasano et al., 2013). *NPAS3* codes for the neuronal PAS domain protein 3 whose expression affects spine density and while its mechanism of action has not yet been resolved, it is suggested that there may be a network composed of NPAS3, NF-κB, BDNF, NGF and VGF (Yang et al., 2016; Pieper et al., 2010). CAPN1 is a calcium-dependent cysteine protease that targets cytoskeletal proteins such as tau and MAPs and is involved in NMDAR activation. It is suggested that development of mature spines is through the NMDAR/Calpain-1 pathway (Wang et al., 2016).

Human-specific genetic variants are implicated in the recycling of NMDAR and AMPAR. NBEA is involved in the endocytic recycling of NMDA receptors likely through a mechanism related to its effect on the distribution of F-actin. Formation, diameter, and maturation of dendritic spines are all affected by NBEA (Niesmann et al., 2011). NSF is an ATPase involved in SNARE disassembly with demonstrated effect on recycling AMPA receptors and potentially NMDA receptors as well. NSF regulates dendritic spine head diameter without modifying spine density or length (Hu and Hsueh, 2024). The apolipoprotein E receptor (ApoE) through the *APOE2*, *APOE3* and *APOE4* variants modify the recycling of GluA1-4 subunits of AMPA receptors and regulate spine density and maturation (Dumanis et al., 2011; Taxier et al., 2022; Dumanis et al., 2009; Neustadtl et al., 2017).

### Genes with effect on WNT signaling

Wnt signaling plays a role in the formation and maturation of dendritic spines and several human-specific genes are involved in this pathway. Isoforms 1 and 2 of DIXDC1 have both been related to dendritic spine density and maturation. Isoform 1 regulates dendritic spine formation by modifying F-actin polymerization through phosphorylation of DIXDC1 by KANK1. On the other hand, isoform 2 has no effect on the cytoskeleton and is thought to exert its function in dendritic spines by Wnt signaling pathways although it has not been confirmed (Kwan et al., 2016). Transcription factor FOXP2 regulates genes involved in synaptic development and plasticity likely through Wnt signaling. (Vernes et al., 2008). *CNTNAP2*, whose function was discussed above, is a FOXP2 target gene that is thought to be negatively regulated by the expression of human FOXP2 (Vernes et al., 2008). Another gene that is negatively regulated by FOXP2 is *MEF2C* and it has been proven that dendritic spine formation is oppositely regulated by FOXP2 and MEF2C (Lazaro et al., 2019; Chen et al., 2016).

FOXP2 is a forkhead transcription factor on human chromosome 7q31 which is known for its importance in the development of speech and language. The gene first attracted attention when an arginine to histidine substitution in the DNA-binding domain was identified as the culprit of an inherited speech/language disorder in a family. It was shown that mice homozygous for an equivalent genetic variation had reductions in cerebellar growth and weight gain, while mice heterozygous for the variation had deficits in motor-skill learning and synaptic plasticity in the striatum and cerebellum (French et al., 2019; Kuo et al., 2023).

Golgi-staining in P12 *Foxp2* ^−/−^ knockout mice showed reductions in the total spine density of striatal projection neurons (SPNs). Foxp2 is targets *Mef2C* and has been shown to negatively regulate dendritic spines and excitatory synapses in hippocampal neurons and cultured striatal neurons. Foxp2 suppresses *Mef2c* through direct DNA binding having specific synaptic and behavioral effects in vocalization of mouse pups (Chen et al., 2016). In zebra finch, FoxP2 reduction caused deficits in vocal during song learning. FoxP2 downregulation in spiny neurons reduced spine density suggesting that FoxP2 might directly or indirectly regulate spine dynamics in brain regions related to song learning phase thereby influencing vocal plasticity (Schulz et al., 2010). The protein is highly conserved across different species; however, the human version has undergone 2 notable amino acid changes since divergence from our common ancestor with the chimpanzee. Both changes are in exon 7 and include a threonine to asparagine change at position 303, and an asparagine to serine change at position 325 (Enard et al., 2002). Of these, it is known that the amino acid substitution at position 325 creates a potential site for phosphorylation by protein kinase C, and also creates a small change in predicted secondary structure of the FOXP2 protein (Enard et al., 2002). This suggests that this amino acid substitution may confer a human-specific functional change for the protein. However, the hypothesis that *FOXP2* underwent recent selection was refuted in a dataset that leveraged genetic diversity panels (Atkinson et al., 2018).

The putative role of human-specific FOXP2 in spoken language may relate in part to its effects on neuronal dendrites and synaptic plasticity. When humanized *FOXP2* was knocked into mice, medium spiny neurons had increased dendritic length compared to wild type mice. The humanized medium spiny neurons also had nearly twice as strong long-term synaptic depression compared to wildtype (Reimers-Kipping et al., 2011). Humanized mice also demonstrated altered vocalizations and reduced dopamine concentrations in several brain regions (Reimers-Kipping et al., 2011). Together, these data suggest that humanized FOXP2 has a strong role in dendritic morphology and synaptic plasticity.

MRI studies have shown that individuals with *FOXP2* gene variations often exhibit volume reductions in several brain regions, including the hippocampus, thalamus, globus pallidus, and caudate nucleus (Liegeois et al., 2016). Functional MRI (fMRI) studies have revealed reduced activation in core language and speech networks in individuals with *FOXP2* variants (Liegeois et al., 2016; Hoogman et al., 2014). Common *FOXP2* variants in the general population have not shown significant effects on brain structure using standard volumetric techniques (Hoogman et al., 2014).

### Orphan genes with yet unclear mechanism related to the dendritic spine

There are some human-specific genes and genetic variants that affect dendritic spinogenesis and maintenance; however, at the current state of knowledge cannot be classified into a known dendritic spine mechanism. GTF2I, PDCD4, SEPTIN7, NLGN4X and androgen receptor (AR) all influence dendritic spine biology through distinct mechanisms suggesting that the human dendritic spine harbors additional layers of regulation. This is consistent with the more expansive proteomic variability of the human dendritic spine and synapse when compared to mice (Italia et al., 2022; Purkey and Dell'Acqua, 2020). GTF2I and PDCD4 are transcription factors that activate the BDNF pathway to regulate the number of dendritic spines. The exact mechanism of these transcription factors in the formation of spines is unknown but is has been proposed that GTF2I regulates downstream PI3K–Akt–mTOR and PDCD4 can act via the AKT-CREB signaling pathway (Li et al., 2021; Borralleras et al., 2015; Cheng et al., 2024). SEPTIN7 phosphorylation promotes its localization in the base of dendritic spines to act as a diffusion barrier to maintain spine components within the dendritic shaft. Other members of the *SEPTIN* family, such as SEPTIN2 and SEPTIN6, were related to dendritic protrusion formation whereas SEPTIN7 was specifically related to mature spine formation without affecting protrusion density (Tada et al., 2007; Yadav et al., 2017).

Androgens and their analogs, which target androgen receptors (AR), have been shown not to increase spine density in the short term but to promote morphological maturation of thin spines to mushroom and to increase the dendritic spine surface area. Testosterone regulates spine maturation through regulation of BDNF and PSD-95 expression in neurons (Chen et al., 2022; Li et al., 2012). Another orphan gene, *NLGN4X,* is an X chromosome linked gene that regulates spine density and maturation: phosphorylation of residue S712 of NLGN4X reduces mushroom spines while the unphosphorylated state of S712 increases spine number (Lehr et al., 2025). SLC6A4 is related to changes in dendritic spine morphology and is a key protein for serotonin uptake in the synaptic cleft (Baron-Mendoza et al., 2021).

Collectively, these genes do not act isolated but converging on a limited number of biological pathways that govern the dendritic spine function. Across the categories, recurrent pathways include (i) remodeling of actin and microtubule cytoskeleton, which determines spine shape and stability; (ii) regulation of Ca^2+^ dynamics and Rho GTPase signaling coupled to rapid morphological plasticity; (iii) trafficking and turnover of postsynaptic glutamate receptors, which define spine strength; and (iv) transcriptional networks–such as Wnt- and FOXP2- related–which coordinate developmental timing and long-term maturation. This convergence suggest that human-specific genetic variation may act by fine-tuning conserved and defined molecular pathways to prolong maturation, increase flexibility and diversify responses of dendritic spines rather than creating novel pathways in the human brain.

## Discussion

We systematically reviewed high confidence human-specific genes to present a snapshot of current knowledge and to highlight their potential contributions to the human dendritic spine as the structural basis for brain function. We integrate the mechanistic insights elucidated mostly in rodent models with human genetics to explore how these human specific genes and variants may modify canonical pathways. Except for the human variants *CHRFAM7A* and *TBC1D*, all genes discussed here have homologs across vertebrate species, and their role in neuronal development including dendritic spine formation have been extensively validated in animal models. While the effect of the human-specific variants remains understudied, it is plausible that these uniquely human genetic changes contribute to dendritic complexity and plasticity observed in the human brain.

Diverse genetic mechanisms are represented in human-specific genes and genetic variants, including segmental duplication-driven genomic rearrangement, gene expansion and fusion genes, retrotransposon-mediated gene evolution and fixed single nucleotide variants (SNVs) as expected (Acuna-Hidalgo et al., 2016). In some cases, genomic rearrangements lead to the expansion of gene families harboring multiple copies, some of which underwent a genetic variation (Soto et al., 2025). This results in complex genetics where the original gene function is altered from its *de novo* copy by a genetic variation, such as in the *SGRAP2* family (Charrier et al., 2012). In others, such as *TBC1D3*, human-specific gene expansion resulted in gene amplification and dosage increase, highlighting gene dosage as a mechanism of added genetic complexity (Hodzic et al., 2006; Guitart et al., 2024).

It is also important to note that some of these human-specific genetic changes are obligatory, while others are facultative, and that these understandings are necessary for effective study and analysis. *FOXP2*, for example, underwent a fixed human-specific genomic variant that led to a human-specific variant of the gene (Enard et al., 2002) which may have contributed to the ability of humans to use language. Analysis of human genomes across all major continents revealed this is a fixed variant in humans, obligatory for normal brain development (Enard et al., 2002; Liegeois et al., 2016). In contrast, *CHRFAM7A* is an example of a facultative human-specific gene. This specific locus is unique in harboring an additional level of genetic complexity due to the existence of two distinct alleles which are in direct or inverted orientation (Szigeti et al., 2020). The allele frequencies are 50% in the US population, thus individuals may have only direct, only inverted or even no *CHRFAM7A* (0.9%) highlighting that *CHRFAM7A* is not obligatory for normal human development (Szigeti et al., 2020). Interestingly, the inverted allele is detected at the highest frequency in East Asia, while the direct allele is most frequent in Africa, suggesting emergence in specific geographic areas and subsequent genetic mixing (Liegeois et al., 2016). As the *CHRFAM7A* alleles are facultative for humans they provide a biallelic human genetic background for brain function and disease risk (Szigeti et al., 2020; Jakimovski et al., 2024).

These observations reveal important gaps in our current knowledge of human-specific variants. The allele frequencies of most of these genes and variants are unknown in the human population, as they have been detected in comparative studies when the human genome from a handful of individuals was compared and contrasted with prehuman primate genomes (154). Assessment of allele frequencies in diverse human populations is needed to determine whether each gene is an obligatory or facultative variant for the human phenotype. Moreover, whether these human specific variants represent gain of function, loss of function, or the emergence of a new function is largely unknown and also requires further characterization.

Surprisingly only a handful of genes have been comprehensively studied in dendritic spine biology, and even fewer have had studies in a human model (human iPSC, human organoid, clinical studies) (Supplementary Table S1 (Lewis et al., 2023; Jang et al., 2023; D'Onofrio et al., 2023; Smogavec et al., 2016; Rodenas-Cuadrado et al., 2014; Nguyen et al., 2020; Lawson-Yuen et al., 2008; Mulhern et al., 2018; Volders et al., 2011; Miura et al., 2021; Jacobsen et al., 2015; Redies et al., 2012; da Silva et al., 2016; Weber et al., 2016; Binder and Nemeroff, 2010; Martin et al., 2018; Takata et al., 2013; Fortea et al., 2024; Heise et al., 2024; Wang X. et al., 2018; Greaves and Rohrer, 2019; Forrest et al., 2018; Zhang et al., 2017; Tahir et al., 2000; Buchholz and Zempel, 2024; Nasser et al., 2020; Rimol et al., 2010; Erdogan et al., 2025; Kraeme et al., 2011; Bergmann et al., 2003; Chabrol et al., 2005; Santos-Reboucas et al., 2014; Jakimovski et al., 2024; Wang Y. et al., 2018; Enard et al., 2002; Liegeois et al., 2016; Hoogman et al., 2014; Kumar et al., 2011; Sugumar et al., 2022; Flynn et al., 2013; Inoue et al., 2023; Einarsdottir et al., 2017; Vilboux et al., 2017; Kunii et al., 2015; Gillentine et al., 2018; Sinkus et al., 2015; Kim et al., 2025; Wu et al., 2012; Misbahuddin et al., 2002; Brancati et al., 2003; Valle-Bautista et al., 2024; Lucena et al., 2010; Deurloo et al., 2019; Liang et al., 2019; Lopez-Tobon et al., 2023; Lerer et al., 2005; Kakinuma et al., 2009; Bocchetta et al., 2023; Toya et al., 2023; Caporale et al., 2024; Pickard et al., 2009; Rossi et al., 2021; Bernier et al., 2014; Guitart et al., 2024; Hayashi et al., 2023; Kozlova et al., 2022; Garret et al., 2020; Suzuki et al., 2021; Unda et al., 2023; Antonarakis et al., 2021; Prufer et al., 2014; Gao et al., 2025; Capelli et al., 2022; Stodberg et al., 2015; Gregory et al., 2019; Hahn and Blakely, 2007; Broer and Gether, 2012; Pezawas et al., 2008; Saitsu et al., 2012; Meng et al., 2023; Pinner et al., 2017; Rogers et al., 2013; Mancini et al., 2020; Thomas et al., 2018; Yu et al., 2017; Song et al., 2022; Sutcliffe et al., 2005; St George-Hyslop et al., 2022; Di et al., 2022) despite the fact that all genes listed except four have already been linked to neurological diseases. To understand and highlight the effect of human specific genes and genetic variants on dendritic spine biology we integrated these variants with canonical dendritic spine pathways that were elucidated in mostly rodent models. The summarized experimental data (Supplementary Table S2) demonstrate that the ancestral genes infer highly specific and dissociable effects on dendritic spines. Several genes, including *APOE*, *ARHGAP15*, and *CRHR1*, primarily influence spine density or their total number, whereas others–such as the *CDH* family and *GRIN3A*–affects spine maturation or morphological features (e.g., length, neck width, area) without altering density or number. *CNTNAP2* knockout mice, for example, exhibit a reduction in dendritic spine density in L2/3 pyramidal neurons, yet show no detectable changes in spine area or synapse length (Gdalyahu et al., 2015). On the contrary, *NSF* knockdown mice reduces the width of dendritic protrusions in neurons while leaving length and density unchained (Hu and Hsueh, 2024). While these experiments do not specifically test the human specific variant, it is plausible that human-specific variants in these genes might selectively modulate distinct dimensions in dendritic spine architecture to influence the final structural and functional state of spines.

The human specific variants will likely align their effect with the ancestral gene refining that in a human specific way. Nearly all genes associated with direct actin regulation have some reported influence in spine maturation or morphology, consistent with the central role of actin in shaping spine structure. Likewise, variants related to RhoGTPases, which can also directly regulate actin remodeling, predominantly affect morphology rather than spine number. This pattern suggests that the human genetic variants of pathways converging on the actin cytoskeleton will likely affect structural remodeling of dendritic spines rather than their *de novo* formation or abundance.

In contrast, variants affecting NMDA receptors converge on calcium-dependent signaling pathways and are more consistently associated with changes in spine density. Approximately 20% of the ionic current through NMDA receptors is carried by calcium empathizing their shared pathways in synaptogenesis and synaptic stabilization (Weaver and Popescu, 2025; Skeberdis et al., 2006). This distinction suggests that NMDA receptor pathways and calcium signaling primarily regulate spine emergence and stabilization. Thus, human genetic variants mapping to these pathways will likely affect spine density and the synapse.

This framework allows us to separate the variants into two functional groups. The first group includes those that directly influence the actin cytoskeleton–mainly variants in actin and Rho GTPase categories–which are expected to affect spine morphology. The second group consist of the variants that influence the cytoskeleton indirectly, such as those variants related to calcium signaling, NMDA-receptor pathways, and Wnt-signaling pathway which are more likely to influence spine density. With this framework in mind, systematic characterization of human-specific variants is needed to understand how they influence the dendritic spine at multiple levels: individually, collectively as obligatory variants that may define the “humanized” dendritic spine, and in interaction with facultative variants that could further modulate it.

The human brain is embedded in a complex and constantly changing environment. The dendritic spine serves as a critical structural substrate for experience-dependent plasticity acting as a physical record of an organism’s interaction with its surroundings. Human-specific gene duplications such as *SRGAP2C* and *TBC1D3* uniquely modulate this process by inducing neotemy-a prolonged state of neuronal immaturity that extends the temporal window for environmental influence. (Charrier et al., 2012). Both genes delay dendritic spine maturation, increasing the proportion of thin and highly dynamic spines while delaying the appearance of stable mushroom spines (Schmidt et al., 2022). Because thin spines are more plastic and responsive to remodeling, their extended period of presence in neurons shapes synaptic connectivity (Schmidt et al., 2022). This extended window of synaptic refinement could facilitate the precise fine-tuning of cortical networks underlying language, abstract reasoning and higher cognitive functions of the human brain, albeit this hypothesis needs to be tested in human clinical studies.

This evolutionary trajectory represents a trade-off, where the selective advantage of cognitive flexibility is intrinsically linked to an increased vulnerability to neurological dysfunction. On one hand, by delaying dendritic spine maturation SRGAPC2 and TBC1D3 facilitate a protracted period of environmental calibration, allowing for the acquisition of complex cultural and linguistic traits. Conversely, the prolonged window for plasticity extends the human brain’s exposure to neurtoxicants and social stressors during critical developmental phases (Liston et al., 2009; McEwen and Morrison, 2013), the environmental factors implicated in neuropsychiatric disorders such as schizophrenia, anxiety, bipolar disorder and as a modifiable risk factor for age related cognitive impairment (Baker et al., 2025). Inherent increased metabolic demand to maintain higher plasticity may overload the neuronal system leading to destabilization potentially explaining why the genomic regions most associated with human-genetic variants significant overlap with schizophrenia susceptibility loci (Supplementary Table S1).

Given these findings, we propose that dendritic spine architecture emerges from a coordinated interaction between signaling systems that regulate spine formation and those that remodel spine structure. Calcium influx and NMDA receptors and related pathways appear to define the temporal window and probability of synapse formation, while downstream regulators, including RhoGTPases and actin-associated pathways, determine the structural stabilization, morphology, and functional integration of those synapses. These human-specific variants could therefore modulate not only the number of synaptic contacts but also their temporal maturation dynamics and stability, ultimately influencing brain efficiency and plasticity. Future studies are needed to elucidate how these pathways interact and how human-specific variants shift the balance toward prolonged refinement phases.

There are important limitations to our study. The list of human-specific genes and genetic variants is ever evolving with the advancement of technologies such as LR sequencing and bioinformatic approaches and pipelines (Soto et al., 2025). We selected a manually curated list to provide a snapshot and identify gaps of knowledge where concentrated efforts are needed. We show that even the most curated list has limited information in model systems and even less in human clinical studies. We utilize artificial intelligence (AI) for first pass results followed by careful manual curation. Although AI can substantially broaden internet search results, no method can yield results completely comprehensive of all existing research. We also identified errors in AI derived conclusions during manual curation of the references and there is also some expected output variation between different AI models. Different large language models are trained on different datasets and employ different algorithms and architecture. Data source and quality might differ as well, as Copilot for example, emphasizes the use of large-scale genomics datasets while other AI models may depend heavily on functional validated data. Thus, each approach has limitations and strengths (Health Care and Life Sciences Blog, 2024). Furthermore, hallucination and bias is inherent in AI models, meaning that they may invent or over-represent associations, leading to the inclusion of irrelevant genes or the exclusion of recently discovered ones. This study mitigated these limitations through careful manual validation of all AI outputs. More AI-based systematic literature reviews will be required to understand which engines are best suited for different topics, and to optimize methods of combining results from different search engines.

For this review, we used the catalog of human-specific genetic variants compiled by Bitar et al. as our starting point (Bitar et al., 2019). This dataset integrates evidence of gene duplications, novel coding regions and structural changes to provide a broader overview of genes with human-specific features. Broader and complementary datasets are evolving, albeit with limited curation. Kuhlwilm and Boeckx (2019), focused primarily on single nucleotide variations (SNVs) in coding regions that differ between modern humans and archaic hominids. Soto et al., meanwhile, are focusing on gene expansions through structural variants (Soto et al., 2025). While the list is ever evolving, even the most curated dataset demonstrates the significant gap of knowledge in functional and clinical characterization of human specific variants.

Current experimental models also present limitations in elucidating human molecular mechanisms within dendritic spines. Most functional and morphological studies on human genetic variants rely on knockout or knockdown mouse models. While these *in vivo* models can resemble the physiological environment, they inherently lack translational relevance due to species-specific differences in gene function, brain organization, and neurodevelopmental timing. Studies on the human-specific genes *CHRFAM7A* and *TBC1D3* are notable exceptions (Szigeti et al., 2023; Dong et al., 2024), having been investigated using human induced pluripotent stem cells (iPSCs). However, current iPSC models, particularly 2D cultures, are restricted by the absence of mature circuits and spatial constraints, lacking the complexity of long-term functional stability and limited dendritic arbors and mature spine morphologies found in human brain. To overcome these challenges, more complex models such as 3D cultures or cerebral organoids are needed. These advanced systems enable the study of human-specific genes and their effects on dendritic spines within organized, short-distance regional circuits.

Parallel to these efforts, human clinical studies with deep phenotyping of brain function using cognitive measures, electrophysiology, structural and functional MRI, and PET scans are needed. In the case of the obligatory human-specific variants, several were identified as phenotype-associated genomic variants (Supplementary Table S1), which validates human-specific function. When the human-specific gene is facultative, such as in the case of *CHRFAM7A*, comparing carriers to noncarriers may reveal how the genetic background may affect brain structure and function.

A full exploration of these human-specific genes and functional characterization in a human model is of great importance. While the human brain evolved to meet new environmental demands, it also altered the fundamental structural basis of the brain, possibly creating new predisposition or vulnerability to neuropsychiatric diseases such as schizophrenia, anxiety, and attention-deficit disorder (Ihnatovych et al., 2024; Kolk and Rakic, 2022). A comprehensive genetic understanding of these human-specific loci, including the genetic mechanism, allele frequency, and the risk they may contribute to neuropsychiatric diseases would facilitate a deeper understanding of the pathophysiology of neuropsychiatric disorders leading to successful therapy development (Makin, 2025; XWPharma Ltd, 2025).

## Supplementary material

SUPPLEMENTARY TABLE S1 Dendritic spine-related human-specific genes and associated phenotypes. Genes implicated in dendritic spine function that exhibit human-specific features, such as gene duplications, deletions, or accelerated regions. It includes details on their genetic human-specific genomic change, allele frequencies, known disease associations and relevant neuroimaging findings.

SUPPLEMENTARY TABLE S2 Effects of human-specific genes on dendritic spine development and function. Roles of selected human-specific genes in pathways influencing dendritic spine formation, morphology or maintenance. It includes hypothesized mechanisms of action (actin cytoskeleton regulation, RhoGTPase signaling, NMDA/AMPA receptor signaling, Wnt signaling or calcium signaling) and key findings in dendritic spines from experimental data in human or animal models.
