# Supplementary material for: The effect of human-specific genetic variants on neuronal spinogenesis
Source: Front Genet. 2026 Jun 3;17:1786287. doi: 10.3389/fgene.2026.1786287 (PMC13271839; doi:10.3389/fgene.2026.1786287)
Supplement: Supplementary file 2 [file DataSheet1.pdf]

Supplementary material

AI Prompt

“Is ______ gene a human-specific gene?

Is it involved in:

1. cytoskeleton function

2. immune function or cancer

3. neuron function

4. cell structure (especially cytoskeleton of neurons or dendritic spine)

5. neuronal pathology or disease”
